# Host-Range Restriction of Vaccinia Virus E3L Deletion Mutant Can Be Overcome *In Vitro*, but Not *In Vivo*, by Expression of the Influenza Virus NS1 Protein

**DOI:** 10.1371/journal.pone.0028677

**Published:** 2011-12-13

**Authors:** Susana Guerra, Fernando Abaitua, Luis Martínez-Sobrido, Mariano Esteban, Adolfo García-Sastre, Dolores Rodríguez

**Affiliations:** 1 Department of Molecular and Cellular Biology, Centro Nacional de Biotecnología, CSIC, Madrid, Spain; 2 Department of Preventive Medicine and Public Health, Universidad Autónoma, Madrid, Spain; 3 Department of Microbiology and Immunology, University of Rochester School of Medicine and Dentistry, Rochester, New York, United States of America; 4 Department of Microbiology, Mount Sinai School of Medicine, New York, New York, United States of America; 5 Division of Infectious Diseases, Department of Medicine, Mount Sinai School of Medicine, New York, New York, United States of America; 6 Global Health and Emerging Pathogens Institute, Mount Sinai School of Medicine, New York, New York, United States of America; National Institute of Environmental Health Sciences, United States of America

## Abstract

During the last decades, research focused on vaccinia virus (VACV) pathogenesis has been intensified prompted by its potential beneficial application as a vector for vaccine development and anti-cancer therapies, but also due to the fear of its potential use as a bio-terrorism threat. Recombinant viruses lacking a type I interferon (IFN) antagonist are attenuated and hence good vaccine candidates. However, vaccine virus growth requires production in IFN-deficient systems, and thus viral IFN antagonists that are active *in vitro*, yet not *in vivo*, are of great value. The VACV E3 and influenza virus NS1 proteins are distinct double-stranded RNA-binding proteins that play an important role in pathogenesis by inhibiting the mammalian IFN-regulated innate antiviral response. Based on the functional similarities between E3 and NS1, we investigated the ability of NS1 to replace the biological functions of E3 of VACV in both *in vitro* and *in vivo* systems. For this, we generated a VACV recombinant virus lacking the *E3L* gene, yet expressing *NS1* (VVΔE3L/NS1). Our study revealed that NS1 can functionally replace E3 in cultured cells, rescuing the protein synthesis blockade, and preventing apoptosis and RNA breakdown. In contrast, *in vivo* the VVΔE3L/NS1 virus was highly attenuated after intranasal inoculation, as it was unable to spread to the lungs and other organs. These results indicate that there are commonalities but also functional differences in the roles of NS1 and E3 as inhibitors of the innate antiviral response, which could potentially be utilized for vaccine production purposes in the future.

## Introduction

Vaccinia Virus (VACV) is a member of the Poxviridae family, a group of large, double-stranded DNA viruses that replicate exclusively in the cytoplasm of the infected host cell [1], [2]. Vaccination with VACV was directly responsible for the successful eradication of smallpox, a devastating disease in man caused by variola virus. The possible re-emergence of variola virus has led to renewed interest in the study of poxvirus pathogenesis using the *in vivo* models that are limited primarily to vaccinia, cowpox, and ectromelia viruses, which do not cause disease in immunocompetent humans [1], [2].

Moreover, the potential use of VACV as a vector for anti-cancer therapies and vaccine purposes has also renewed interest in understanding the basis of poxvirus pathogenesis and attenuation. Recombinant viruses lacking a type I interferon (IFN) antagonist are attenuated and hence good vaccine candidates [3]. However, efficient vaccine virus growth requires production in IFN-deficient systems. Hence, the identification of viral IFN antagonists that are active *in vitro*, yet do not contribute to virus virulence *in vivo* are of great value.

Poxviruses contain a large array of genes which are used to evade host immune responses and contribute to pathogenesis [4], [5], [6]. VACV encodes multiple proteins that interfere with complement regulatory proteins, cytokines and chemokines, toll-like receptors (TLRs), signal transduction pathways, and apoptosis. [6]. One of the VACV proteins with strong inhibitory activity of IFN-induced pathways is E3 [7], [8], [9]. VACV mutants lacking E3 (VVΔE3L) only replicate in IFN-incompetent cell systems [9], are non-pathogenic in mice, yet provide protection against wild-type virus challenge [10], [11]. E3 has two functional domains, one located at the N-terminus, that is required for its nuclear localization and Z-DNA binding activity, and which is also involved in the direct inhibition of protein kinase R (PKR), and the dsRNA-binding domain at the C-terminus, required for IFN-resistance and for the broad host range phenotype of the virus [10], [12], [13].

The E3 protein represses the host cell antiviral response by multiple mechanisms, including inhibition of the two well-characterized IFN-inducible enzymes PKR and 2′-5′-oligoadenylate synthetase (2′-5′OAS), both being activated by dsRNA [8], [14], [15]. Activation of these two proteins triggers a global inhibition of protein synthesis, which leads to the induction of apoptosis and an effective blockade of viral replication [16]. Upon binding to dsRNA, PKR mediates phosphorylation of the alpha subunit of the eukaryotic protein synthesis initiation factor (eIF-2α) leading to a translational block. On the other hand, upon stimulation, 2′-5′OAS generated products activate an endogenous endoribonuclease (RNase L), which cleaves cellular and viral RNAs [17]. Therefore, the ability of E3 to inhibit activation of these enzymes is crucial for the maintenance of the cellular translational function, which is required for active viral replication. E3 also blocks induction of IFN-α/β through inhibition of phosphorylation of the IFN regulatory transcription factors 3 (IRF-3) and 7 (IRF-7) [18], [19], and prevents nuclear factor κB (NF-κB) activation [20]. Furthermore, the E3 protein binds to the protein encoded by IFN-stimulated gene 15 (*ISG15*), thereby blocking its antiviral activity [21]. The E3 deleted mutant virus, VVΔE3L, is restricted for replication in many tissue culture systems, but replicates in double knock-out PKR/RNase L [19] or ISG15 deficient murine cells [21], and in PKR deficient human cells [22]. Recently it has been described that the formation of antiviral granules, due to the phosphorylation of eIF-2α by PKR, also imposes a restriction on VVΔE3L replication in murine cells [23].

In addition to E3, VACV encodes other proteins that also inhibit the IFN action. They either prevent IFN binding to its natural receptor (soluble type I and II IFN-binding proteins) or block the IFN-signalling cascade or IFN-induced antiviral state within infected cells [24]. One of these proteins, B19, is a soluble IFN-α/β receptor that is expressed very early in the infection. Viruses lacking *B19R* gene were shown to be attenuated in both intranasally and intracranially infected mice [25], supporting the importance of B19 in pathogenesis. Another VACV protein involved in the ablation of IFN signalling is B8, a soluble IFN-γ receptor, which is also expressed early in infection [26]. However, the deletion of the *B8R* gene from the VACV genome did not attenuate pathogenesis in a mouse model [27].

Influenza virus is a segmented negative-stranded RNA virus causing significant respiratory infections in humans. This virus expresses a non-structural protein in infected cells, the NS1 protein, which counteracts the IFN response. Although this protein does not share significant amino acid identity with the VACV E3 protein, its functional properties are remarkably similar to those of the E3 protein.

The N-terminal domain of influenza A virus NS1 protein binds to dsRNA preventing the dsRNA-mediated activation of 2′-5′OAS and RNase L [28]. As E3, NS1 binds directly to PKR and inhibits its kinase activity [29], [30], blocks the induction of IFN-α/β through inhibition of IRF3 and IRF7 phosphorylation [31] and prevents NF-κB [32], janus kinase (JNK) and activating transcription factor 2 (ATF-2) activation [33]. An influenza virus mutant lacking NS1 (ΔNS1) is restricted in replication in IFN-competent systems, but replicates and induces disease in mice lacking either PKR or STAT1, a transcription factor required for IFN signalling. In addition to NS1, the influenza viral polymerase complex has been also shown to exhibit an inhibitory activity on IFN-β promoter activation [34]. Moreover, the PB1-F2 90 amino acid protein expressed from the PB1 gene of some influenza A viruses has also been shown to suppress IFN-stimulated genes in vitro and in vivo [35].

Taking the differences and similarities between E3 and NS1 into account, here, we wanted to investigate the ability of influenza A NS1 (Puerto Rico/8/34 strain) to replace VACV E3 in both *in vitro* and *in vivo* systems. To this end, we generated a recombinant VACV lacking E3 but expressing NS1, VVΔE3L/NS1. Our study revealed that NS1 can functionally replace E3 in cultured cells, inhibiting RNA degradation, protein synthesis blockade and the apoptotic process, being all of these biochemical effects caused in cells infected with the E3L deletion mutant, VVΔE3L [36].

Although the recombinant VVΔE3L/NS1 was able to grow in VVΔE3L non-permissive cultured cells, this virus was unable to replicate in tissues of infected animals. In fact, our studies revealed that mice infected with VVΔE3L/NS1, like those infected with VVΔE3L, do not show signs of infection, even when high doses of these viruses were used, while animals infected with wild-type VACV showed severe weight loss and die a few days after inoculation. These results indicate that there must be functional differences between NS1 and E3 as inhibitors of the innate antiviral response regulated by the IFN system, which are not apparent in tissue culture, but are highlighted during infection in an animal model.

## Materials and Methods

### Cells, viruses, and infections

HeLa (human epithelial cervical cancer cells, ATCC number CCL-2™), BSC40 (african green monkey kidney cells, ATCC number CRL-2761), BHK21 (baby hamster kidney cells, ATCC number CCL-10) and NIH-3T3 (mouse embryo fibroblast cells, ATCC number CRL-1658) were grown in Dulbecco's modified minimal essential medium (DMEM) containing penicillin (100 U/ml) and streptomycin (100 µg/ml) and 10% fetal calf serum (FCS) (Sigma). Wild-type VACV (strain Western Reserve (WR)) and the recombinant virus expressing the influenza A virus NS1 protein were grown in monkey BSC-40 cells, purified through two 45% (w/v) sucrose cushions, and titrated in triplicate by plaque assay on BSC40 cells [36]. The VACV E3 deletion mutant in which the *E3L* gene was fully deleted by being replaced by the *E. coli* β-galactosidase coding gene has been previously described [37], and will be named in this study as VVΔE3L for simplicity. VVΔE3L virus was grown in BHK21 cells and titrated by immunostaining [36]. In brief, BHK21 cells grown in 6 well plates were infected with serial dilutions of viral extracts and at 18 hours post-infection (h.p.i.) cells were fixed and permeabilized with Methanol∶Acetone (1∶1) for 2 min. Then cells were washed with PBS and incubated with a polyclonal antibody against VACV proteins [38] diluted 1∶1000 in phosphate-buffered saline (PBS) containing 3% FCS (PBSF). After 90 min incubation at room temperature, cells were washed twice with PBS and a peroxidase-conjugated goat anti-rabbit serum diluted 1∶1000 in PBSF was added. 90 min later the wells were washed twice with PBS and 1 ml of a solution containing 1 mg/ml of diaminobenzydene (DAB), 0.03% hydrogen peroxide and 0.03% nickel sulphate diluted in PBS, was added to each well. The reaction was stopped with PBS when the foci of infected cells were visible. For tissue culture infections, near-confluent monolayers of cells were mock-infected or infected with the different viruses diluted in supplemented DMEM to the indicated multiplicity of infection (MOI). After 1 h of adsorption at 37°C, virus and medium were removed and replaced with fresh DMEM containing 2% FCS. Infected cells were incubated at 37°C until the indicated times p.i.

### Engineering of the VACV recombinant viruses

The DNA fragment corresponding to the gene coding for the NS1 protein from the Puerto Rico/8/34 (H1N1) strain of influenza A virus (accession number P03496) was cloned into the *Sma* I site of the VACV insertion vector pJR101 [39]. The resulting plasmid, pJRNS1, contains the *NS1* gene under the control of a VACV synthetic early/late promoter, Pse/l, [40], the *E. coli β-glucoronidase* marker gene under the control of the VACV P7.5 early/late promoter, and all these sequences flanked by regions from VACV hemagglutinin (HA) coding gene (*A56R* gene). Insertion of the *NS1* gene into VVΔE3L genome was achieved by *in vivo* recombination between the HA flanking sequences present in the pJRNS1 vector and the HA locus in the virus genome, which results in the partial deletion of this viral gene. Specifically, recombinant viruses VVΔE3L/ΔHA and VVΔE3L/NS1 were obtained by infection of BHK21 cells with the VVΔE3L mutant at 0.01 plaque forming units per cell (PFU/cell) and transfection with the empty pJR101 or pJRNS1 plasmids respectively in presence of lipofectamine (Invitrogen). Cell cultures were harvested at 48 h.p.i and the recombinant viruses were selected after plaque assay by the addition of 5-bromo-4-chloro-3-indolyl-β-D- glucuronic acid (X-Gluc) substrate to the agar overlay. Similarly, cells infected with WR virus were transfected with empty pJR101 plasmid to obtain the VV/ΔHA control virus.

### Quantitative real-time RT-PCR

Total RNA was isolated from purified VACV, VVΔE3L or VVΔE3L/NS1-infected (5 PFU/cell) or mock-infected HeLa cells with Ultraspect-II RNA (Biotecx), following the manufacturer's instructions. One µg of RNA was reverse-transcribed using SuperScript (Invitrogen) with oligo-dT as a primer. A 1∶40 dilution of the RT reaction mixture was used for quantitative PCR. Primers and probe sets used to amplify IFN-β (Hs01077958-s1), ISG15 (Hs00192713-m1), IL-6 (Hs00174131-m1), IFN-α (Hs01652729-s1) and ATF-3 (Hs00231069-m1), were purchased from Applied Biosystems. RT-PCR reactions were performed according to Assay-on-Demand, optimized for TaqMan Universal PCR MasterMix, No AmpErase UNG, as described [41]. All samples were assayed in triplicate. Threshold cycle (Ct) values were used to plot a standard curve in which Ct decreased in linear proportion to the log of the template copy number. The correlation values of standard curves were always >99%.

### SDS-PAGE and Western-blot analysis

Following VACV infection, cells were lysed in disruption buffer (0.5% Triton X-100, 50 mM KCl, 50 mM NaCl, 20 mM Tris-HCl [pH 7.5], 1 mM EDTA, 10% glycerol, 1×Complete protease inhibitor cocktail (Roche), 25 mM β-glycerophosphate, 1 mM Na_3_VO_4_). Lysates were separated by SDS-PAGE with the same amount of total protein being loaded into each lane and then transferred onto nitrocellulose membranes. Immunoblots were blocked for 1 h in PBS containing 0.05% Tween 20 (PBST) and 5% non-fat dry milk, washed in PBST, and incubated at 4°C overnight with different primary antibodies in PBST and 1% non-fat dry milk. The antibodies used in this study were rabbit polyclonal sera raised against a VACV recombinant virus expressing β-galactosidase [38], against VACV E3 (a generous gift of B. Jacobs, Arizona University, Arizona, USA), against VACV HA (a generous gift from J. Ichihasi, Niigata University, Niigata, Japan) or against NS1 (a generous gift of J. Ortín, Centro Nacional de Biotecnología, Madrid, Spain). Blocking and primary antibody incubation for total eIF-2α (Santa Cruz Biotechnology), pS^35^ eIF-2α (Invitrogen), and PARP (Cell Signaling) were performed according to instructions provided by the manufacturer. Subsequently, membranes were washed and incubated for 2 h with horseradish peroxidase-conjugated goat anti-mouse or anti-rabbit immunoglobulin G (Sigma), and bound antibodies were detected with the ECL Western-blotting detection reagent (GE Healthcare).

### Viral inoculation of mice and sample collection

C57/BL-6 wild-type, PKR^−/−^ [42] or ISG15^−/−^ [43] mice (6 to 10 week-old) were inoculated intra-nasally (i.n.) in 25 µl of PBS with VV/ΔHA, VVΔE3L/ΔHA or VVΔE3L/NS1 at 10^7^, 5×10^6^ or 5×10^5^ PFU/mouse as indicated. All animals were weighed immediately before virus inoculation and the following days; mice were also monitored daily for survival. Animals with >30% loss of body weight were sacrificed. The results shown represent the mean values obtained in 3 independent experiments ± standard deviation. The overall significance of the curves (*P* values) was determined using a two-tailed t test assuming non-equal variance. In all the cases we obtained *P*<0.01. Inoculated animals were sacrificed at various times post-inoculation and organs were removed aseptically, weighed, homogenized in DMEM (0.1 mg of tissue/ml), and subsequently assayed in triplicate for viral yield by standard plaque assay in BSC40 cells for VV/ΔHA or VVΔE3L/NS1, or by immunostaining assay in BHK21 cells for VVΔE3L/ΔHA. All animals were handled in strict accordance with good animal practice as defined by the relevant national, international, and/or local animal welfare bodies, and with the Spanish Royal Decree (RD 1201/2005). All animal work was approved by the Ethical Committee of Animal Experimentation (CEEA-CNB) of the Centro Nacional de Biotecnología (CNB-CSIC).

### Histochemistry

Formalin-fixed lungs from infected mice were resected, sectioned and stained with hematoxilin and eosin as previously described [21]. Tissue sections were visualized using a Leica DMRXA microscope and images captured with the DC100 imaging system (Leica). Three different biological samples were analyzed.

## Results

### Generation of the virus VVΔE3L/NS1

**Figure 1:**
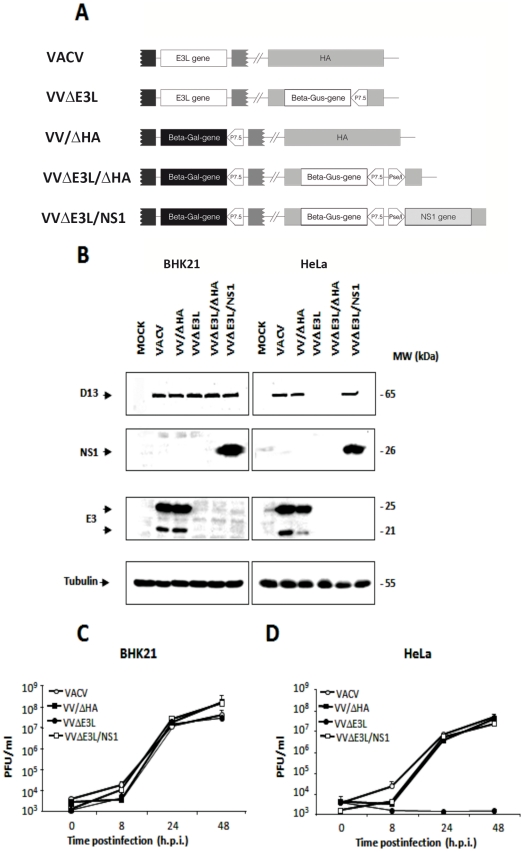
Generation, characterization, and growth properties of VACV recombinants. **A. Schematic representation of the VACV recombinants used for this study.** The mutant virus VVΔE3L was generated by replacing the VACV *E3L* gene by the *β-galactosidase* (beta-gal) marker gene [37]. The influenza A (Puerto Rico/8/34) virus *NS1* gene was introduced into the HA coding region (*A56R* gene) of VVΔE3L virus together with the *E. coli β-glucuronidase* (beta-gus) marker gene to allow the selection of the VVΔE3L/NS1 recombinant virus. Control viruses VV/ΔHA and VVΔE3L/ΔHA were generated by introducing the *β-glucuronidase* gene into the HA locus of VACV and VVΔE3L genomes, respectively. The VACV P7.5 and Pse/l promoters are indicated. **B. Analysis of E3 and NS1 protein expression by recombinant viruses.** Extracts from BHK21 (left panels) or HeLa (right panels) cells mock-infected (MOCK) or infected (MOI 5) for 24 h with VACV, VV/ΔHA, VVΔE3L, VVΔE3L/ΔHA or VVΔE3L/NS1 were separated by SDS-PAGE. Proteins transferred to nitrocellulose membranes were detected with antibodies against VACV E3 and influenza A virus NS1 proteins. *E3L* gene directs the synthesis of two proteins of 25 kDa and 20 kDa, of which the latter was previously suggested to be a product of internal initiation. As a control for viral infection an antibody against the VACV D13 protein was also used. Tubulin was detected as a protein loading control. **C and D. Growth curves of mutant viruses in permissive BHK21 (C) or non-permissive HeLa cells (D).** Infected cells (0.01 PFU/cell) were harvested at different times p.i. and virus yields were determined by plaque assay (VACV, VV/ΔHA, VVΔE3L/NS1) or by immunostaining (VVΔE3L). Results represent the mean ± the standard deviation of three independent experiments.

VVΔE3L is unable to replicate in HeLa cells, however, it has been shown that viral replication can be restored by expressing other viral dsRNA-binding proteins such as the reovirus σ3 [37], the rotavirus NSP3 [44] and the parapoxvirus Orf virus (OV) E3 homolog [45]. To investigate whether the expression of NS1 protein form influenza A virus (Puerto Rico/8/34 (H1N1) strain) by VVΔE3L could rescue the biological defects of this mutant, we generated a recombinant virus where the NS1 coding sequence was inserted into the HA locus (*A56R* gene) of the VVΔE3L genome. Control viruses based on wild-type VACV or VVΔE3L in which the HA coding gene was also partially deleted (VV/ΔHA and VVΔE3L/ΔHA, respectively) were also constructed. Figure 1A shows a schematic representation of the viruses used in this study.

The expression of the NS1 protein by VVΔE3L/NS1 was verified by Western-blot analysis of infected BHK21 cells, a cell line that is permissive to VVΔE3L replication most likely due a deficient IFN-mediated antiviral response [36]. It was also confirmed that this virus does not express any of the two forms (25 kDa and 21 kDa) of the E3 protein, as opposed to VACV and VV/ΔHA (Figure 1B). The reactivity of the same samples with an antibody against the VACV D13 (65 kDa) protein served as an infection control and indicated that BHK21 cells were similarly infected by all viruses used. An anti-tubulin antibody was used for protein loading control.

The growth kinetics of VVΔE3L/NS1 was compared to those of VVΔE3L, VACV and VV/ΔHA in BHK21 cells. For this, monolayers of BHK21 cells were infected at low MOI (0.01 PFU/cell) and at the indicated times p.i. cells and media were collected together and assayed in triplicate for viral yield quantitation. VVΔE3L/NS1 has recovered the ability to form plaques in BSC40 cells (not shown), therefore, as for VACV and VV/ΔHA, virus yields were determined by plaque assay, whereas VVΔE3L viral titers were determined by immunostaining. Similar yields were obtained with the four viruses in the permissive BHK21 cells at the different time points analyzed (Figure 1C).

### VVΔE3L/NS1 overcomes the characteristic blockade of VVΔE3L to replicate in HeLa cells

Mutant vaccinia viruses lacking E3 display a replication-defective phenotype in many human cell lines, including HeLa cells [36]. The main function of E3 in cell culture has been shown to be the inhibition of dsRNA-dependent PKR activity [7]. Upon activation through dsRNA binding, PKR phosphorylates the Ser51 residue of the eIF-2α translation initiation factor [46]. This leads to the inhibition of both host and viral protein synthesis, thereby inhibiting viral replication. The replication of VVΔE3L can be partially rescued in HeLa cells in which PKR expression is suppressed [47]. Since influenza NS1 protein is also known to prevent PKR activity [48], [49] we next wanted to analyze the phenotype of VVΔE3L/NS1 in HeLa cells, non-permissive for VVΔE3L. At high MOI (5 PFU/cell), expression levels of the NS1 protein in VVΔE3L/NS1-infected HeLa cells were similar to those in the infected BHK21 (Figure 1B). Moreover, the expression of VACV D13 protein in HeLa cells infected with VVΔE3L/NS1 was similar to that observed in cells infected with wild-type VACV or VV/ΔHA viruses; however it clearly diminished to undetectable levels in VVΔE3L- and VVΔE3L/ΔHA- infected cells (Figure 1B).

**Figure 2:**
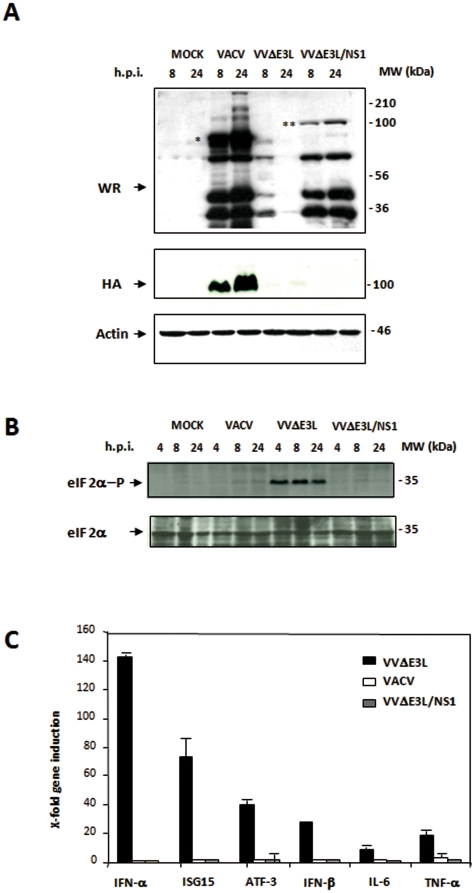
VVΔE3L/NS1 blocks eIF-2α phosphorylation and proinflammatory cytokine production in infected HeLa cells. **A. Viral protein expression.** HeLa cells were mock-infected (MOCK) or infected with VACV, VVΔE3L or VVΔE3L/NS1 (5 PFU/cell). Cells were collected at the indicated times p.i., and equal amounts of total protein from cell extracts were separated by SDS-PAGE, transferred to nitrocellulose, and treated with a polyclonal antibody against a VACV recombinant virus that expresses the *β-galactosidase* gene. An anti-VACV HA antibody was used to confirm the lack of expression of this protein by the recombinant viruses. Actin was used as a protein loading control. The position of HA in VACV infected cells is indicated by an asterisk. The position of β-galactosidase protein observed in VVΔE3L/NS1 is denoted by two asterisks. **B. eIF-2α phosphorylation levels during VACV, VVΔE3L or VVΔE3L/NS1 infection.** HeLa cells were mock-infected (MOCK) or infected with VACV, VVΔE3L or VVΔE3L/NS1 (5 PFU/cell). Cells were collected at the indicated times p.i., and equal amounts of total protein from cell extracts were fractionated by SDS-PAGE, transferred to nitrocellulose and incubated with antibodies to total eIF-2α or eIF-2α phosphorylated at Ser51. **C. Relative expression levels of genes involved in the immune response determined by real time RT-PCR.** HeLa cells were mock-infected (MOCK) or infected with VACV, VVΔE3L or VVΔE3L/NS1 (5 PFU/cell) and processed for real time RT-PCR at 6 h.p.i. The name of each gene product and the data values obtained in RT-PCR are indicated. X-Fold gene induction (x-axis) was quantified by real time RT-PCR in three independent experiments. Standard deviation is indicated.

To study viral growth efficiency, monolayers of HeLa cells were infected at 0.01 PFU/cell with VACV, VV/ΔHA, VVΔE3L or VVΔE3L/NS1, and cells were harvested at different times p.i. and viral titers were determined as mentioned above. As expected, VVΔE3L virus did not replicate in HeLa cells (Figure 1D). However, introduction of the *NS1* gene into the virus genome rescued its ability to replicate in these cells. Similar viral replication curves were observed after VACV, VV/ΔHA or VVΔE3L/NS1 infection. As detected by Western-blot with an anti-VACV polyclonal antibody, viral protein accumulation at 8 and 24 h.p.i. was also increased in VVΔE3L/NS1-infected cells as compared to cells infected with VVΔE3L (Figure 2A). Differential bands between wild-type VACV- and VVΔE3L/NS1-infected cells observed in the Western-blot correspond to the HA protein (denoted with an asterisk), as confirmed by the reactivity with an anti-HA specific antibody, and the β-galactosidase protein (denoted with two asterisks), expressed by wild-type VACV- and VVΔE3L/NS1, respectively. These results indicate that viral protein synthesis and viral growth are rescued in the presence of NS1. As it has been previously described [50], the translational blockade observed in VVΔE3L-infected HeLa cells coincided with the phosphorylation of eIF-2α (Figure 2B). No eIF-2α phosphorylation was observed in VACV- or VVΔE3L/NS1-infected HeLa cells. Therefore these findings show that in the context of VACV infection NS1 is able to block the eIF-2α phosphorylation mediated by PKR, restoring viral protein synthesis. Next, we investigated whether VVΔE3L/NS1 is resistant to IFN treatment. Infection with VV/ΔHA and VVΔE3L/NS1 viruses in BHK21 cells pretreated with different doses of IFN-β (from 0 to 1000 U/ml) indicated that both viruses exhibit similar resistance to IFN inhibition, whereas VVΔE3L is highly sensitive (data not shown). Overall, the results obtained above indicate that NS1 is able to restore to VVΔE3L the capacity to replicate in HeLa cells and the IFN resistance phenotype.

### NS1 blocks the induction of immunomodulatory molecules and activation of IFN pathways in cultured cells infected with VVΔE3L/NS1

VVΔE3L virus was previously shown to elicit high expression of specific immunomodulatory molecules such as TNF-α, IFN-β, IFN-α, IL-6 and ISG15 in HeLa cells [51]. We investigated if the presence of NS1 was sufficient to inhibit the upregulation of these molecules. To that end, we evaluated the mRNA levels of TNF-α, IFN-β, IFN-α, IL-6 and ISG15 in VACV-, VVΔE3L- or VVΔE3L/NS1-infected HeLa cells by real-time RT-PCR and compared them to those in mock-infected cells. During VVΔE3L infection high levels of TNF-α, IFN-β, IFN-α, IL-6 and ISG15 mRNA were detected, which is in accordance with previous publications [51]. However, infection with VACV or VVΔE3L/NS1 prevented induction of those mRNAs (Figure 2C). It has been described that E3 protein can suppress a diverse array of cytokines, produced through both PKR-dependent and PKR-independent pathways [52]. IL-6 and IFN-β expressions are completely PKR-independent, however induction of TNF-α requires only PKR-dependent NF-κB activation [52]. Our results revealed that expression of NS1 in the context of VACV infection suppresses the pro-inflammatory signal transduction pathways in a similar way as the E3 protein.

### NS1 inhibits apoptosis and prevents RNA degradation that characterizes VVΔE3L infections

**Figure 3:**
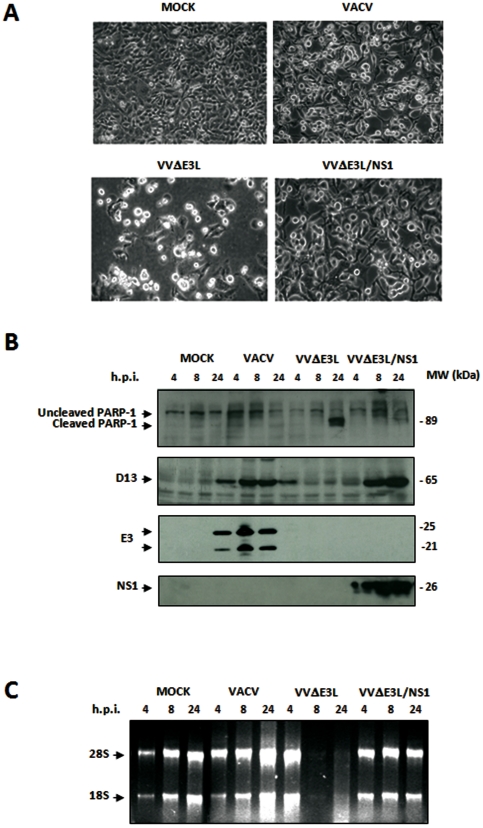
NS1 expression by VACV mutants prevents apoptosis and rRNA degradation. **A. Morphological changes in HeLa cells mock-infected or infected with VACV, VVΔE3L and VVΔE3L/NS1 (5 PFU/cell).** Cell morphology was examined at 16 h.p.i by phase-contrast microscopy. **B. Time course of PARP-1 cleavage during VACV, VVΔE3L or VVΔE3L/NS1 infection.** HeLa cells were mock-infected (MOCK) or infected with VACV, VVΔE3L or VVΔE3L/NS1 (5 PFU/cell). At the indicated times p.i. cells were harvested and total proteins were separated by SDS-PAGE, transferred to nitrocellulose and immunoblotted with anti-PARP-1 antibody. An 89 kDa PARP-1 cleavage product is observed at 24 h.p.i in VVΔE3L infected cells. The expression of VACV D13L and E3 and recombinant NS1 proteins was examined as control of viral infection. **C. rRNA degradation.** Total rRNA was isolated from HeLa cells mock-infected (MOCK) or infected with VACV, VVΔE3L or VVΔE3L/NS1 (5 PFU/cell) at indicated times p.i. Each sample was analyzed by electrophoresis and subsequent staining with ethidium bromide.

Because it has been demonstrated that VVΔE3L infection triggers programmed cell death and RNase L-mediated RNA degradation in HeLa cells [7], we wanted to know if NS1 expression could replace E3 in these inhibitory functions. Apoptosis is mediated by activation of caspase-8 or -9, leading to induction of effector caspases-3 and -7, which cleave specific substrates, including poly (ADP-ribose) polymerase-1 (PARP-1). This enzyme catalyzes the formation of poly (ADP-ribose) polymers on acceptor proteins involved in the maintenance of chromatin structure. The presence of an 89 kDa PARP-1 cleavage product indicates the activation of the apoptotic cascade [53]. We monitored apoptosis by comparing cellular morphological changes in HeLa cells infected with wild-type VACV, VVΔE3L and VVΔE3L/NS1 and assessing cleavage of PARP-1 at different times p.i. We also examined ribosomal rRNA degradation as an indication of RNase L activation. The morphological signs of apoptosis observed in VVΔE3L-infected HeLa cells at 24 h.p.i. were not evident after infection with VVΔE3L/NS1 (Figure 3A). In addition, as VACV, VVΔE3L/NS1 was able to prevent the characteristic cleavage of PARP-1 observed in VVΔE3L-infected cells at 24h.p.i. (Figure 3B). Similarly, rRNA degradation was only observed in VVΔE3L-infected cells (Figure 3C). These findings suggest that NS1 is able to prevent apoptosis and RNase L activity, thereby preventing host rRNA degradation.

### NS1 cannot substitute E3 *in vivo*

To determine if the introduction of the *NS1* gene into VVΔE3L genome alters the attenuated phenotype of the virus *in vivo*, we set out to infect mice with the different mutant viruses. To ensure that all mutant viruses behaved phenotypically similar in human and mouse cells, we infected monolayers of mouse NIH-3T3 cells at 0.01 PFU/cell with VACV, VV/ΔHA, VVΔE3L or VVΔE3L/NS1, harvested at different times p.i. and measured viral titers. As expected, similar to what occurs in human cells, VVΔE3L virus did not replicate in NIH-3T3 cells (Figure S1). In contrast, VVΔE3L/NS1 was able to replicate to the same level that VACV and VV/ΔHA (Figure S1). This indicates that the mutant viruses exhibit the same phenotype in human and mouse cells.

**Figure 4:**
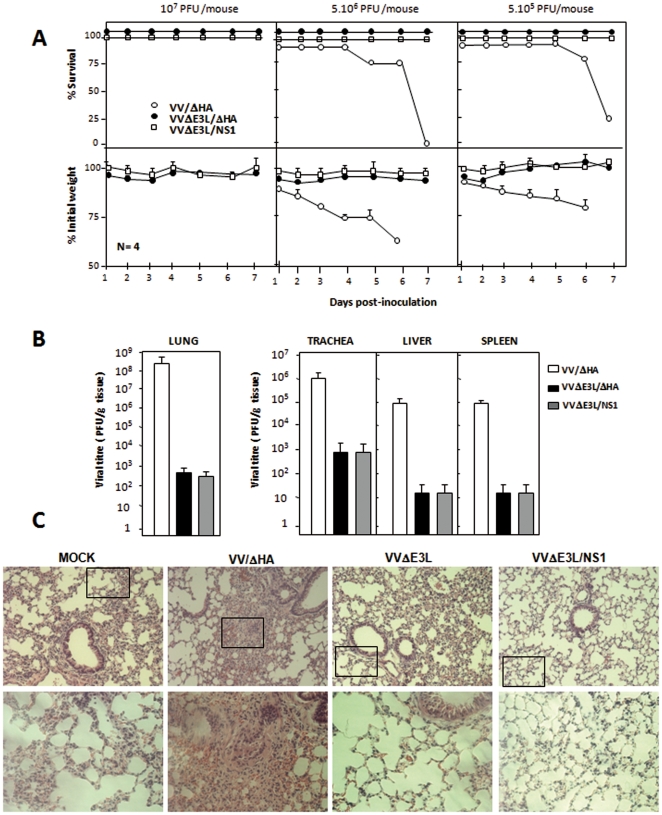
The expression of influenza virus NS1 protein does not modify the attenuated phenotype of VVΔE3L *in vivo*. **A.** C57/BL6 mice were infected i.n. with three doses of VVΔE3L/ΔHA or VVΔE3L/NS1, (10^7^, 5×10^6^ or 5×10^5^ PFU/mouse) and two doses of VV/ΔHA (5×10^6^ or 5×10^5^ PFU/mouse). Weight and survival rates were monitored. Survival curves are shown in the upper panels. The percentage of weight loss of each animal with respect to the initial weight at the starting of the experiment was calculated, and the mean values of four animals per group are represented (lower panels). The error bars represent standard deviation. **B.** Quantitative analysis of viral replication in the lung, trachea, liver, and spleen of VV/ΔHA, VVΔE3L/ΔHA or VVΔE3L/NS1 infected mice (5×10^6^ PFU/mouse) at 3 days p.i. The yields of infectious virus in the different tissues from four animals per group were determined by plaque assay for VV/ΔHA and VVΔE3L/NS1 or by immunostaining for VVΔE3L/ΔHA. The results represent the mean values of 3 independent experiments ± standard deviation. *P* values from a two-tailed t test assuming non-equal variance were determined. In all the cases we obtained *P*<0.01. **C.** Hematoxylin and eosin staining of histological sections of lung tissues from C57/BL6 mice mock-infected or infected i.n. with 5×10^6^ PFU/mouse of VV/ΔHA, VVΔE3L/ΔHA or VVΔE3L/NS1 at 3 days p.i. Upper panels show representative fields, and lower panels represent enlarged areas of fields shown in the upper panels (indicated by the square). Three different biological samples were analyzed.

Next, C57/BL-6 mice were infected i.n. with three challenge doses of VVΔE3L/ΔHA or VVΔE3L/NS1 (10^7^, 5×10^6^ or 5×10^5^ PFU/mouse) or the two lower doses (5×10^6^ or 5×10^5^ PFU/mouse) of VV/ΔHA. Infected mice were scored for weight loss and mortality over a period of one week as prominent indicators of viral pathogenesis. In contrast to VV/ΔHA infection, which caused drastic body weight loss and severe signs of illness, infection of mice with VVΔE3L/ΔHA or with the recombinant VVΔE3L/NS1 did not cause any obvious disease, even at the highest dose used (Figure 4A). Mice infected with 5×10^6^ PFU/mouse of VV/ΔHA started dying at 5 days p.i., and by day 7th all mice had died. In contrast, all mice infected with VVΔE3L/NS1 virus survived. This result revealed that introduction of the *NS1* gene into the backbone of VVΔE3L does not modify the attenuated phenotype of the E3L-deleted virus in mice.

In the mouse model, i.n. inoculation of wild-type VACV results in an acute infection of the lung followed by subsequent spread of the virus to visceral organs. To determine viral dissemination in the animal we analyzed viral titers in homogenates of trachea, lung, liver and spleen obtained at day 3 after infection with the different viruses. We found VV/ΔHA virus in the trachea, from where the virus had progressed to the lungs, liver and spleen of infected animals. Viral titers were >1000-fold higher in all tissues from VV/ΔHA infected animals than in those from animals infected with the VVΔE3L/ΔHA control virus (Figure 4B). Consistent with the survival data, NS1 expression did not rescue viral replication (Figure 4B). Hematoxylin and eosin stained histological sections of lung tissue from animals infected with the different viruses (5×10^6^ PFU/mouse) were examined at 3 days p.i. Lung sections obtained from VVΔE3L/ΔHA- or VVΔE3L/NS1-infected mice showed no inflammatory cells infiltrating the lung parenchyma. In contrast, VV/ΔHA-infected mice presented severe inflammation with alveolar wall thickening and infiltration of inflammatory cells (Figure 4C; insets).

Since the VVΔE3L/NS1 was not pathogenic in wild-type mice, we reasoned that the virus cannot counteract effectively some of the IFN-induced pathways responsible for mounting the antiviral response. To test if either PKR and/or ISG15 proteins were responsible for the *in vivo* restriction of this virus, we infected PKR^−/−^ [42] and ISG15^−/−^ [43] mice with the mutant viruses. VVΔE3L/NS1 was neither lethal in mice lacking PKR (Figure S2), nor in ISG15^−/−^ mice (Figure S3). In both knock-out mouse strains no signs of pathogenesis were detected after the infection with VVΔE3L/NS1, whereas loss of weight and animal death were observed among VACV infected mice (Figures S2 and S3).

Since the VVΔE3L/NS1 virus grows to high titers in tissue culture, yet is attenuated *in vivo*, it could be of interest for future vaccine development. To investigate if the VVΔE3L/NS1 virus still elicits immune responses that protect from wild-type VACV challenge, we pre-immunized wild-type mice with VVΔE3L/ΔHA or VVΔE3L/NS1 by i.n. route and subsequently challenged them with 2×10^7^ PFU/mouse of wild-type VACV. VVΔE3L/ΔHA- or VVΔE3L/NS1-immunized mice did neither lose weight nor developed other signs of illness (Figure S4), indicating that both groups of animals were protected similarly against a lethal VACV challenge. These findings demonstrate that although VVΔE3L/NS1 replicates poorly *in vivo*, it is able to confer protection to challenge with VACV, similar to what occurs with VVΔE3L [54]. However, since VVΔE3L/NS1, in contrast to VVΔE3L, grows to high titers *in vitro*, it may hold a promising strategy for vaccine development.

## Discussion

Since the IFN-induced cellular antiviral response is the primary defense mechanism against virus infections, many viruses have developed a number of strategies to counteract IFN-dependent pathways, and specifically to avoid the deleterious effects of the PKR and 2′-5′ oligoadenylate synthetase/RNase L system. E3 and NS1, from poxvirus and orthomyxovirus, respectively, are both dsRNA-binding proteins highly specialized in blocking cellular defense mechanisms, interfering with these pathways at different levels. These viral inhibitors are normally expressed from the onset of infection to maintain the IFN system inactive until the virus cycle is completed. Elimination of these IFN inhibitors from these viruses generally has a severe impact on virus replication and pathogenesis. VVΔE3L is a virus that only replicates in IFN-incompetent systems [19], is nonpathogenic in the mouse model, and provides protection against a wild-type VACV virus challenge [54].

Here, we describe that the replacement of *E3L* gene with *NS1* resulted in a recombinant virus, VVΔE3L/NS1, that replicated as efficiently as wild-type VACV in cells in culture but was debilitated in pathogenesis in the animal model. These results suggest that in the context of VACV, NS1 is able to block, with the same efficacy as E3, the IFN-based antiviral defense that operates in the VACV *in vitro* infection. One of the main functions of E3 in cell culture has been shown to be the inhibition of dsRNA-dependent PKR activity [7], and the consequent phosphorylation of the eIF-2α translation initiation factor [46]. In the absence of E3, eIF-2α phosphorylation leads to the inhibition of host and viral protein synthesis, thereby inhibiting VVΔE3L replication, and inducing apoptosis. The replication of VVΔE3L and induction of apoptosis can be both partially rescued in HeLa cells in which PKR expression is suppressed [47]. The absence of eIF-2α phosphorylation in VVΔE3L/NS1-infected HeLa cells correlates with the rescue of viral protein synthesis and prevention of apoptosis, indicating a possible inhibition of PKR after NS1 expression. This observation is in concordance with previous results using other systems that show that NS1 prevents the activation of PKR [48], [49]. In addition, VVΔE3L/NS1 is not only able to grow in HeLa cells, but it is also resistant to IFN treatment to the same levels as VACV. Elegant studies have been performed to identify those E3 regions involved in dsRNA binding necessary to mediate the VACV IFN resistance phenotype and the ability to replicate in HeLa cells [55]. In these experiments Shors et *al.* demonstrated that recombinants containing an *E3L* gene with deletions of 37 (VVE3LΔ37N) or 83 (VVE3LΔ83N) amino acids from its N-terminus were IFN resistant and able to replicate in HeLa cells, indicating that those regions have no effect on dsRNA binding activity or PKR inhibition [55]. However, recombinant VACV containing an *E3L* gene deletion of 26 amino acids from its C-terminus (VVE3LΔ26C) or a point mutation at glycine 164 that completely abrogates dsRNA binding activity [13], were both sensitive to the effects of IFN and unable to replicate in HeLa cells. The result obtained here suggest that in infected cultured cells NS1 complements the functions conferred by the E3 C-terminus, hence allowing VVΔE3L/NS1 replication in HeLa cells and conferring IFN resistance.

Despite the complementation observed *in vitro*, expression of NS1 by VVΔE3L/NS1 does not contribute to increase the pathogenicity of the *E3L* deletion mutant. Although it has been shown that the presence of the entire E3 is required for pathogenesis [10] [12], Langland et *al.* showed that there are different behaviors between several *E3L* deletion mutants viruses (VVΔE3L, VVE3LΔ26C, VVE3LΔ83N) regarding the induction of proinflammatory cytokines [51]. They speculate that the rate of expression of host inflammatory genes induced during infection with these mutants and VACV (VVΔE3L>VVE3LΔ26C>VVE3LΔ83N>VACV) is inversely proportional to the virulence associated with these viruses, indicating that these pathways and inflammatory genes could be responsible for limiting the replication of some of these mutant viruses [51]. However, VVΔE3L/NS1 is able to block proinflammatory gene expression and, nonetheless, is highly attenuated *in vivo*. Although we cannot discard the contribution of proinflammatory cytokines in diminishing VACV pathogenesis in the absence of E3, our results suggest that they do not play a crucial role.

The E3 molecule is a conserved feature amongst orthopoxviruses. Vijaysri *et al.* [56] showed that the expression of the OV E3 homologue was able to complement the deletion of VACV *E3L* gene *in vitro* but not *in vivo* [56]. These authors generated chimeric viruses and demonstrated that the N-terminal domain of OV E3 could substitute the function of the N-terminal domain of VACV E3 *in vivo*. The N-terminal domains from vaccinia, Orf, lumpyskin, swinepox, and yaba-like disease viruses have sequence similarity to a family of Z-DNA binding proteins of defined three-dimensional structure and the results obtained by these authors suggest that there is also functional conservation between VACV and OV E3 N-terminal domains. In contrast, the C-terminal domain of OV E3 could not substitute the VACV counterpart. Since the C-terminal domain of E3 is involved in dsRNA binding, these results suggest that OV E3 may bind dsRNA differently in comparison to VACV E3. The *in vivo* phenotype of VVΔE3L/NS1 suggests that the absence of the unique N-terminal Z-DNA binding domain of E3 and/or the presence of a different dsRNA domain could be responsible for the lack of virulence.

On the other hand the *in vivo* replication blockade of VVΔE3L/NS1 appears to be PKR and ISG15 independent since the virus was neither pathogenic in PKR and ISG15 knockout mice. Hence, we reasoned that additional IFN-induced pathways are effectively mounting an antiviral response against this virus *in vivo*.

Overall, these results would suggest that since NS1 fully restores the functionality of E3 in cultured cells, but E3 is still required for VACV virulence, probably E3 exerts additional function(s) essential for its virulence *in vivo* that could not be covered by NS1.

One of the additional functions encoded by E3 that NS1 probably lacks, would be the ability to inhibit cytosolic dsDNA sensing pathways, which are activated after VACV infection due to its cytoplasmic replication. One of the molecules involved in these pathways is the DNA-dependent activator of IFN regulatory factors (DAI). As mentioned before, the E3 N-terminus contains a Z-DNA-binding domain, which shows high amino acid homology to the corresponding domain of DAI [57]. The finding that the substitution of the E3 N-terminus by the dsDNA binding domain from DAI or adenosine deaminase 1 (ADAR1), another Z-DNA-binding protein, does not affect VACV virulence, suggests that dsDNA binding functions in the E3 N-terminus regulate VACV pathogenesis [58]. However, recently it has been demonstrated that the ability of E3 to block signaling in response to dsDNA maps entirely to the C-terminal dsRNA-binding domain, indicating that the binding of the E3 N-terminus to cytoplasmic dsDNA is not responsible of the virulence regulating function [59]. Therefore, the function of the N terminus in virulence still remains unclear [9] [45]. The proposed ability of E3 N-terminus and other Z-DNA-binding proteins to regulate additional pathways involved in the host response, [60], as is the cellular transcription [51], could be a particular characteristic of E3, and probably an essential requirement to allow a virulent phenotype in the *in vivo* infection.

In the context of influenza infection NS1 is an essential requirement to abolish the IFN activity and to achieve virulence. However, in addition to NS1, the influenza viral polymerase complex has been also shown to exhibit an inhibitory activity on IFN-β promoter activation [34]. More recently, the PB1-F2 90 amino acid protein expressed from the *PB1* gene of some influenza A viruses has also been shown to suppress IFN-stimulated genes *in vitro* and in mice [35]. We can speculate the possibility of a synergistic function of the *PB1* gene product together with NS1. In this regard, to further investigate the specific contribution of these factors in the blockade of the IFN system *in vivo* it could be interesting to study the effect of the co-expression of *NS1* and *PB1* genes in the VACV context regarding the restoration of the virulent phenotype. Also, studying the *in vivo* phenotypic effect of the substitution of NS1 by E3 could help to define more specifically the functional similarities and differences between these two important immunomodulator proteins.

On the other hand, despite the highly attenuated phenotype of the VVΔE3L/NS1 virus, preimmunization of mice with this virus confers protection against a lethal challenge with wild-type VACV, indicating that this virus is able to elicit a strong immune response. In conclusion, even though NS1 can functionally substitute E3 in cultured cells, in mice E3 is required for VACV virulence. This result suggests that E3 exerts additional roles required for VACV pathogenesis that are absent in NS1 protein. Apart from being a valuable tool to study the role of *E3L* gene in the virulence of poxviruses, this recombinant construct may prove to be a promising vector for use in recombinant vaccines and anticancer therapy.

## Supporting Information

Figure S1 **VVΔE3L/NS1 is able to grow in murine cells.** Growth curves of VACV, VV/ΔHA, VVΔE3L, and VVΔE3L/NS1 in NIH/3T3 cells. Infected cells (0.01 PFU/cell) were harvested at different times p.i. and virus yields were determined by plaque assay for VACV, VV/ΔHA or VVΔE3L/NS1 or by immunostaining for VVΔE3L or VVΔE3L/ΔHA. Results represent the mean ± the standard deviation of three independent experiments.
(TIF)

Figure S2 **PKR deficiency does not contribute to VVΔE3L/NS1 pathogenesis.** Weight and survival rates of PKR^−/−^ and their wild-type counterparts C57/BL6 mice infected i.n. with VACV, VVΔE3L or VVΔE3L/NS1 at 5×10^6^ or 5×10^5^ PFU/mouse. The percentage of weight loss of each animal was established by comparing with its starting weight before infection, and error bars indicate the standard deviation for each group of 5 mice. *P* values from a two-tailed t test assuming non-equal variance were calculated. In all the cases we obtained *P*<0.01.
(TIF)

Figure S3 **ISG15 deficiency does not contribute to VVΔE3L/NS1 pathogenesis.** Weight and survival rates of ISG15−/− and their wild type counterparts C57/BL6 mice infected i.n. with VACV or VVΔE3L/NS1 at 5×10^6^ or 5×10^5^ PFU/mouse. The percentage of weight loss of each animal was established by comparing with its starting weight before infection, and error bars indicate the standard deviation for each group of 5 mice. *P* values from a two-tailed t test assuming non-equal variance were determined. In all the cases we obtained *P*<0.01.
(TIF)

Figure S4 **Infection with VVΔE3L/NS1 protects mice from lethal WR challenge.** **A.** Quantitative analysis of the survival and loss of weight C57/BL6 mice primed i.n. with 5×10^5^ PFU/mouse of VVΔE3L/ΔHA or VVΔE3L/NS1 and challenged by i.n. route with VACV at 2×10^7^ PFU/mouse. The percentage of weight loss of each animal was established by comparing with its starting weight before infection and error bars indicate the standard deviation for each group of 5 mice. *P* values from a two-tailed t test assuming non-equal variance are indicated, *P*<0.01.
(TIF)
