# Supplementary figures and images for: Host-Range Restriction of Vaccinia Virus E3L Deletion Mutant Can Be Overcome In Vitro, but Not In Vivo, by Expression of the Influenza Virus NS1 Protein
Source: PLoS One. 2011 Dec 13;6(12):e28677. doi: 10.1371/journal.pone.0028677 (PMC3236761; doi:10.1371/journal.pone.0028677)

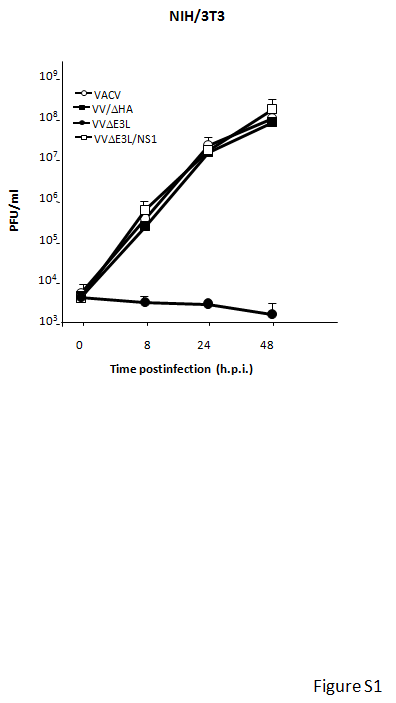

Supplement: Figure S1 — VVΔE3L/NS1 is able to grow in murine cells. Growth curves of VACV, VV/ΔHA, VVΔE3L, and VVΔE3L/NS1 in NIH/3T3 cells. Infected cells (0.01 PFU/cell) were harvested at different times p.i. and virus yields were determined by plaque assay for VACV, VV/ΔHA or VVΔE3L/NS1 or by immunostaining for VVΔE3L or VVΔE3L/ΔHA. Results represent the mean ± the standard deviation of three independent experiments. (TIF) [file pone.0028677.s001.tif]

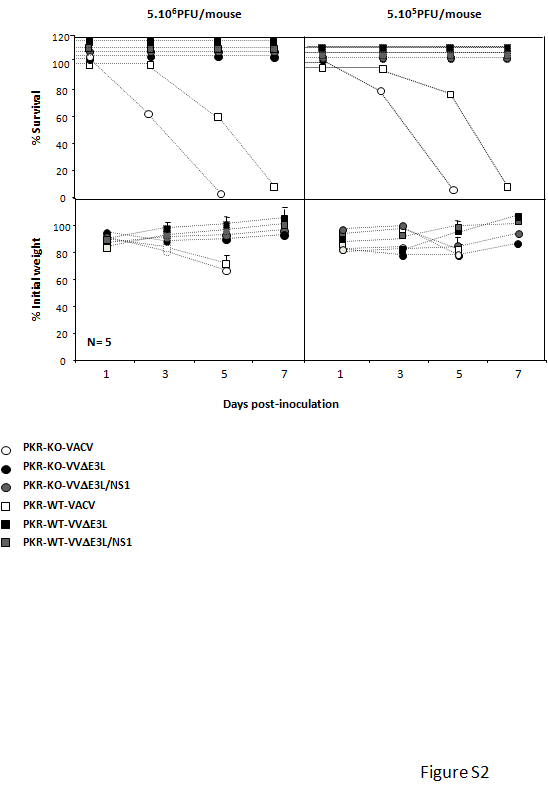

Supplement: Figure S2 — PKR deficiency does not contribute to VVΔE3L/NS1 pathogenesis. Weight and survival rates of PKR−/− and their wild-type counterparts C57/BL6 mice infected i.n. with VACV, VVΔE3L or VVΔE3L/NS1 at 5×10^6^ or 5×10^5^ PFU/mouse. The percentage of weight loss of each animal was established by comparing with its starting weight before infection, and error bars indicate the standard deviation for each group of 5 mice. P values from a two-tailed t test assuming non-equal variance were calculated. In all the cases we obtained P<0.01. (TIF) [file pone.0028677.s002.tif]

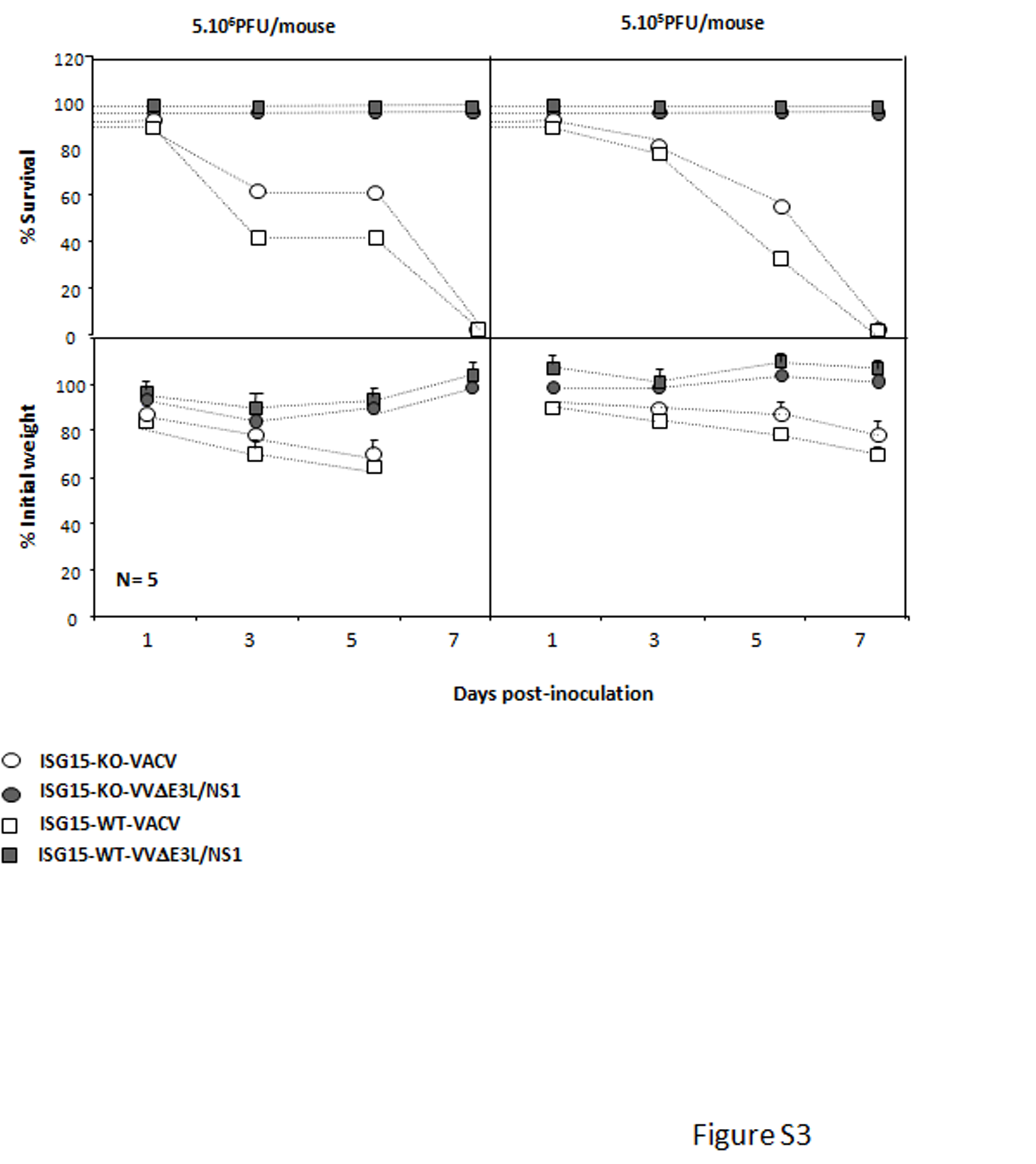

Supplement: Figure S3 — ISG15 deficiency does not contribute to VVΔE3L/NS1 pathogenesis. Weight and survival rates of ISG15−/− and their wild type counterparts C57/BL6 mice infected i.n. with VACV or VVΔE3L/NS1 at 5×10^6^ or 5×10^5^ PFU/mouse. The percentage of weight loss of each animal was established by comparing with its starting weight before infection, and error bars indicate the standard deviation for each group of 5 mice. P values from a two-tailed t test assuming non-equal variance were determined. In all the cases we obtained P<0.01. (TIF) [file pone.0028677.s003.tif]

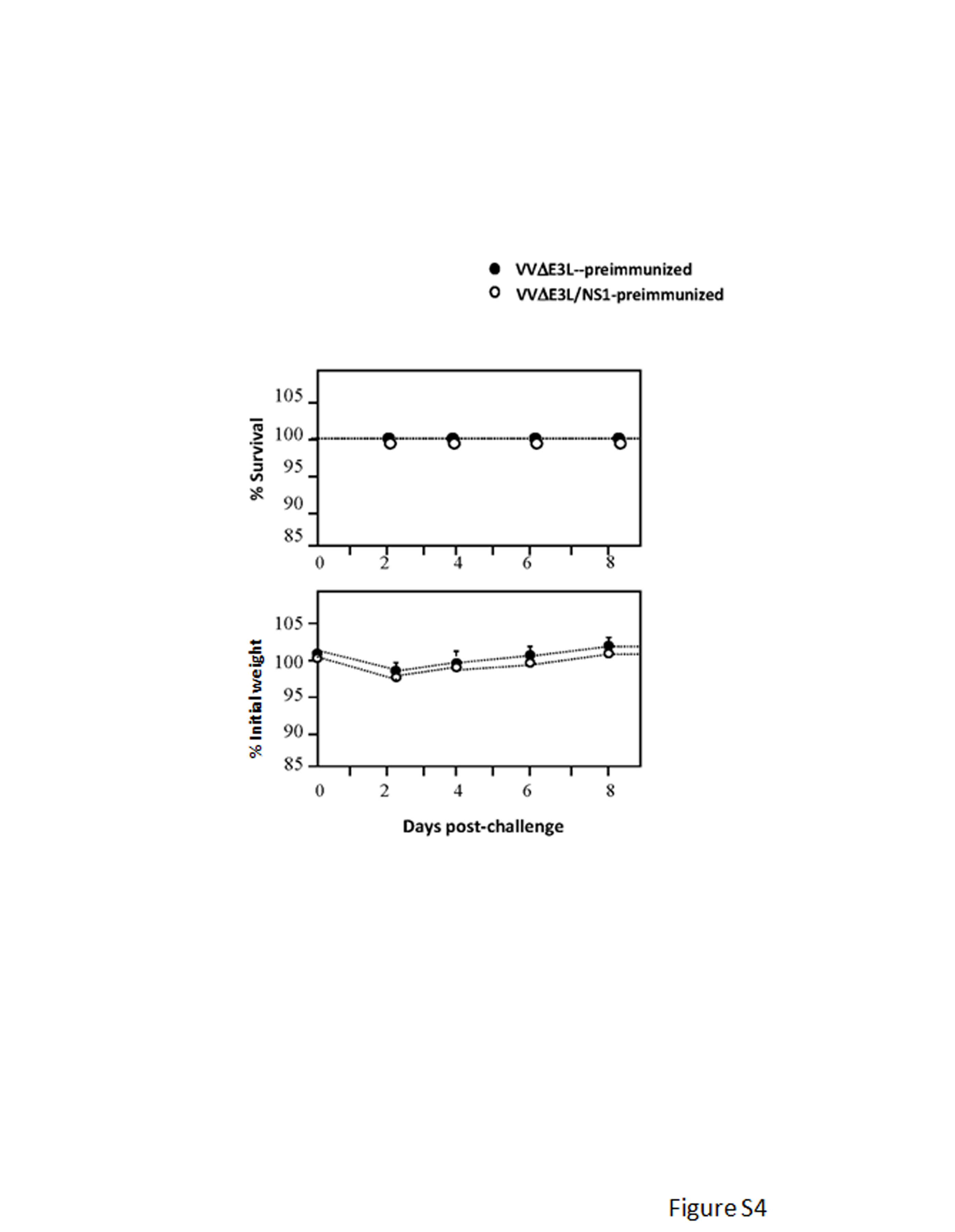

Supplement: Figure S4 — Infection with VVΔE3L/NS1 protects mice from lethal WR challenge. A. Quantitative analysis of the survival and loss of weight C57/BL6 mice primed i.n. with 5×10^5^ PFU/mouse of VVΔE3L/ΔHA or VVΔE3L/NS1 and challenged by i.n. route with VACV at 2×10^7^ PFU/mouse. The percentage of weight loss of each animal was established by comparing with its starting weight before infection and error bars indicate the standard deviation for each group of 5 mice. P values from a two-tailed t test assuming non-equal variance are indicated, P<0.01. (TIF) [file pone.0028677.s004.tif]
